# Supplementary material for: Assessing the energy transition in China towards carbon neutrality with a probabilistic framework
Source: Nat Commun. 2022 Jan 10;13:87. doi: 10.1038/s41467-021-27671-0 (PMC8748502; doi:10.1038/s41467-021-27671-0)
Supplement: Supplementary file 1 — Supplementary Information [file 41467_2021_27671_MOESM1_ESM.pdf]

Supplementary Information

Assessing the energy transition in China towards carbon neutrality with a probabilistic framework

Shu Zhang, Wenying Chen*

Institute of Energy, Environment and Economy, Tsinghua University, 100084 Beijing, PR China

Number of pages: 24

Number of tables: 3

Number of figures: 18

Number of notes: 1

***Corresponding author**

Wenying Chen

Email: chenwy@tsinghua.edu.cn

Supplementary Table 1. The assumptions of social and economic drivers in the China-TIMES-MCA model.

Drivers	2010	2015	2020	2025	2030	2035	2040	2045	2050
GDP (billion 2005 USD)	3,838	5,591	7,388	9,656	12,033	14,995	18,686	21,663	25,113
Population (million)	1,339	1,375	1,412	1,430	1,450	1,444	1,435	1,419	1,395
Urbanization (%)	49.9%	56.1%	63.9%	67.9%	70.8%	73.5%	75.7%	77.4%	78.8%
Primary industry (%)	9.3%	8.4%	7.7%	6.5%	5.6%	4.9%	4.3%	3.8%	3.4%
Secondary industry (%)	46.5%	40.8%	37.8%	35.5%	33.6%	31.5%	29.5%	27.5%	25.7%
Tertiary industry (%)	44.2%	50.8%	54.5%	58.0%	60.8%	63.6%	66.2%	68.7%	70.9%

Notes: The data in the table for 2010 and 2015 are the final validated data from the National Bureau of Statistics, the data for 2020 are the preliminary accounting statistics from the National Bureau of Statistics, and the data for 2025 and beyond are based on the projections domestic experts. GDP = gross domestic product.

Supplementary Table 2. Statistical summary of uncertain input parameters

Parameters	Distribution	Min	1-quartile	Mid	3-quartile	Max	Mean	SD
**Bio Cap**	Log-normal	0.700	0.926	1.000	1.080	1.430	1.007	0.117
**BECCS Cost**	Log-normal	0.700	0.925	1.000	1.080	1.430	1.007	0.117
**Solar Cap**	Normal	0.700	0.935	1.000	1.070	1.300	1.000	0.097
**Solar Cost**	Log-normal	0.700	0.926	1.000	1.080	1.430	1.007	0.117
**Storage Cost**	Log-normal	0.700	0.926	1.000	1.080	1.430	1.007	0.117
**Wind Cap**	Normal	0.800	0.956	1.000	1.040	1.200	1.000	0.065
**Wind Cost**	Log-normal	0.800	0.953	1.000	1.050	1.250	1.003	0.072
**Thermal CCS Cost**	Log-normal	0.700	0.926	1.000	1.080	1.430	1.007	0.117
**Hydrogen Cost**	Log-normal	0.700	0.926	1.000	1.080	1.430	1.007	0.117
**Industry CCS Cost**	Log-normal	0.700	0.926	1.000	1.080	1.430	1.007	0.117
**Nuclear Cap**	Normal	0.700	0.935	1.000	1.070	1.300	1.000	0.097
**Nuclear Cost**	Log-normal	0.700	0.926	1.000	1.080	1.430	1.007	0.117
**Price Elasticity**	Normal	0.700	0.935	1.000	1.070	1.300	1.000	0.097
**Carbon Budget**	Uniform	240.0	255.0	270.0	285.0	300.0	270.0	17.34

Supplementary Table 3. Technology investment cost projections for the intermediate case in the China-TIMES-MCA model.

Investment cost (USD/kW)		2020	2025	2030	2035	2040	2045	2050
Thermal power without CCS[1,2]	Biomass & coal co-combustion	716	698	681	664	664	664	664
	Ultra-supercritical coal-fired power	550	547	545	542	540	538	535
	IGCC coal-fired power	1,454	1,324	1,206	1,098	1,000	911	829
	Nature gas steam turbine power	550	550	550	550	550	550	550
Thermal power with CCS[1-4]	Biomass & coal co-combustion retrofit	1,021	947	878	835	794	774	755
	Biomass combustion	2,128	1,916	1,726	1,554	1,400	1,261	1,136
	Ultra-supercritical coal-fired power	1,062	1,031	1,000	970	941	913	886
	Oxygen enriched coal-fired power	1,062	1,039	1,017	995	974	953	933
	IGCC coal-fired power	2,107	1,868	1,655	1,467	1,300	1,152	1,021
	NGCC gas-fired power	921	912	904	897	897	897	897
Nuclear[2,5,6]	PWR nuclear power	1,995	1,946	1,897	1,850	1,805	1,805	1,805
	HTGR nuclear power	2,857	2,571	2,286	2,171	2,057	1,943	1,829
Renewables[2,5]	PV solar power	754	637	538	454	383	324	324
	Offshore wind power	1,980	1,859	1,746	1,640	1,540	1,446	1,358
	Onshore wind power	1,095	1,056	1,019	983	948	915	882
	Small hydro power	1,312	1,312	1,312	1,312	1,312	1,312	1,312
	Large hydro power	1,066	1,066	1,066	1,066	1,066	1,066	1,066
Energy storage[2,7,8]	Compressed air energy storage	824	741	666	629	594	560	529
	Flow cell battery energy storage	1,064	818	782	668	570	486	415
	Flywheel energy storage	273	238	208	190	174	159	146
	Lithium battery energy storage	5,235	4,352	2,712	2,271	1,892	1,577	1,293
	Lead battery energy storage	1,198	1,072	725	662	599	536	505
	Pumped hydro storage	885	821	762	742	723	704	686
Hydrogen[1,8]	Water electrolysis	1,138	837	691	539	420	327	255

Notes: CCS = carbon capture and storage; IGCC = integrated gasification combined cycle; NGCC = natural gas combined cycle; PWR = pressurized water reactor; HYGR = high temperature gas-cooled reactor; PV = photovoltaic.

Supplementary Notes 1

The assumptions of the distributions are based on the actual variations in parameters In this paper, a log-normal distribution is used for the cost-related parameters, and expresses the effect of percentage changes in costs in economic terms. Since there are no universally accepted national carbon budget allocation results, we chose a wide range of cumulative carbon budgets and used a uniform distribution to represent the effect of different cumulative carbon budgets on the pathways. For other parameters, we chose normal distribution to reflect the parameter uncertainty in a balanced way. When performing uncertain case generation, we fixed the median of all parameters (except Carbon Budget) to 1 and used the result as a basis for other scenarios.

The uncertain case generator used in our study was developed by Sandia National Laboratories for the generation of multivariate samples with a constrained randomization termed Latin hypercube sampling (LHS). The generation of these samples is based on user-specified parameters that dictate the characteristics of the generated samples, such as the type of sample (LHS or random), sample size, number of samples, correlation structure of input variables, and type of distribution specified for each variable. The following distributions are built into the program: normal, lognormal, uniform, log-uniform, triangular, and beta. In addition, the samples from the uniform and log-uniform distributions may be modified by changing the frequency of sampling within subintervals, and a subroutine which can be modified by the user to generate samples from other distributions (including empirical data) is provided. The actual sampled values are used to form vectors of variables commonly used as input computer models for sensitivity and uncertainty analyses. The software code, documentation and input data can be found at Zenodo (https://doi.org/10.5281/zenodo.5717886)[9].

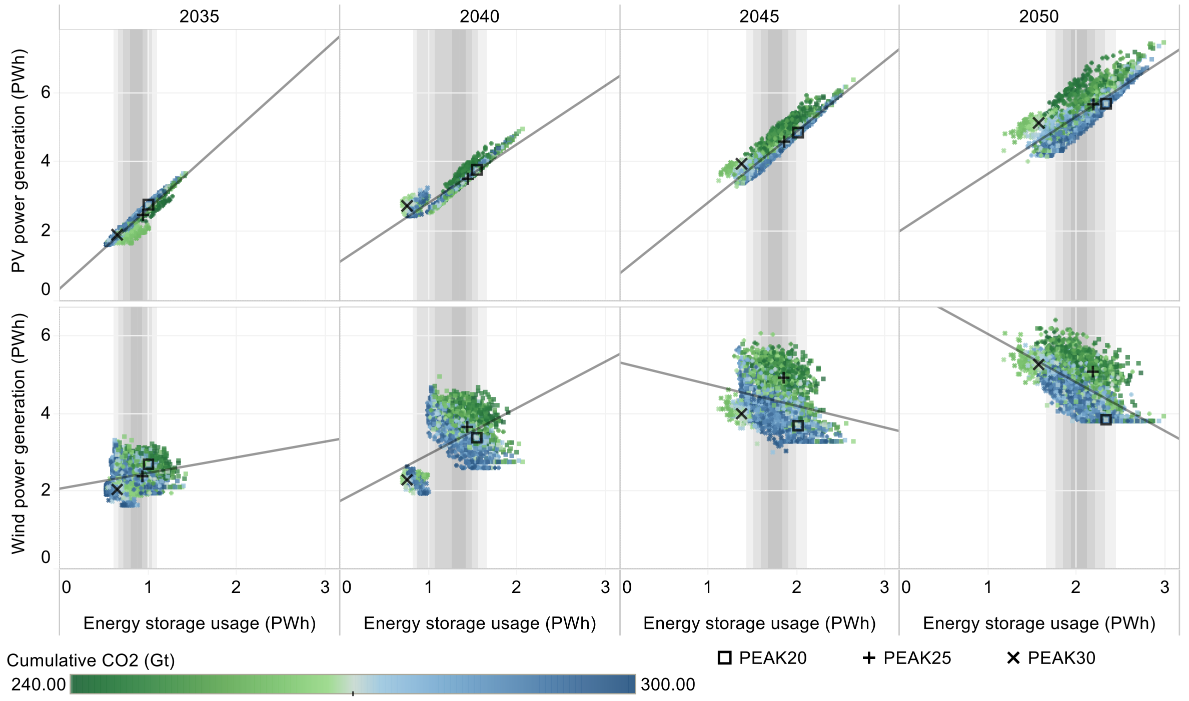

Supplementary Fig. 1. Coupling relationship among energy storage usage, wind power generation and PV power generation. The shadows of different levels indicate the positions of the ten quantiles of energy storage. The divergent colour from blue to green reflects the increasing stringency of the cumulative carbon budget. The cumulative CO_2 parameter corresponds to the absolute value of China's cumulative carbon budget for 2010-2050. The PEAK20, PEAK25, and PEAK30 scenarios are denoted with square, plus, and multiplication signs, respectively. Black symbols represent the intermediate cases for PEAK20, PEAK25, and PEAK30, respectively. PV = photovoltaic.

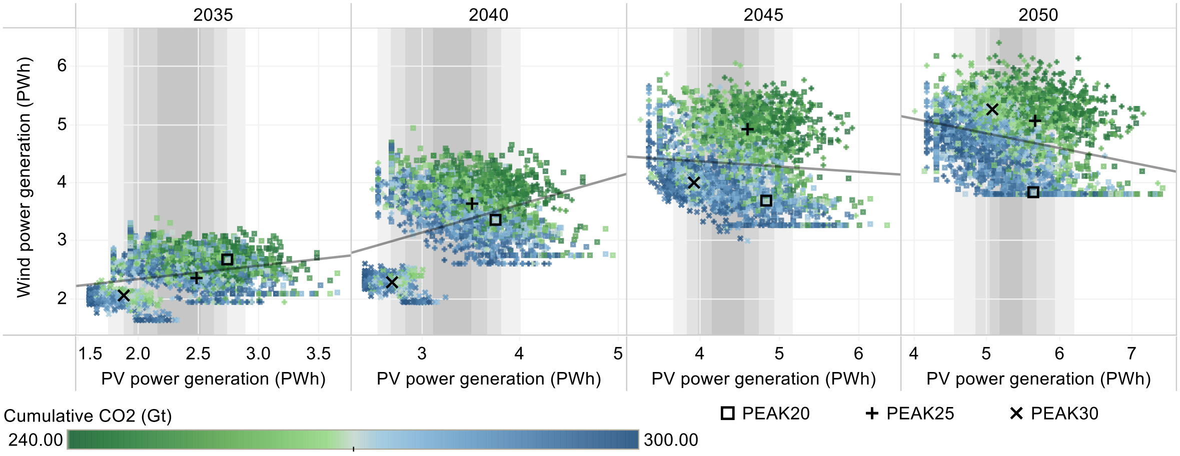

Supplementary Fig. 2. Coupling relationship between wind power generation and PV power generation. The shadows of different levels indicate the positions of the ten quantiles of PV power generation. The divergent colour from blue to green reflects the increasing stringency of the cumulative carbon budget. The cumulative CO_2 parameter corresponds to the absolute value of China's cumulative carbon budget for 2010-2050. The PEAK20, PEAK25, and PEAK30 scenarios are denoted with square, plus, and multiplication signs, respectively. Black symbols represent the intermediate cases for PEAK20, PEAK25, and PEAK30, respectively. PV = photovoltaic.

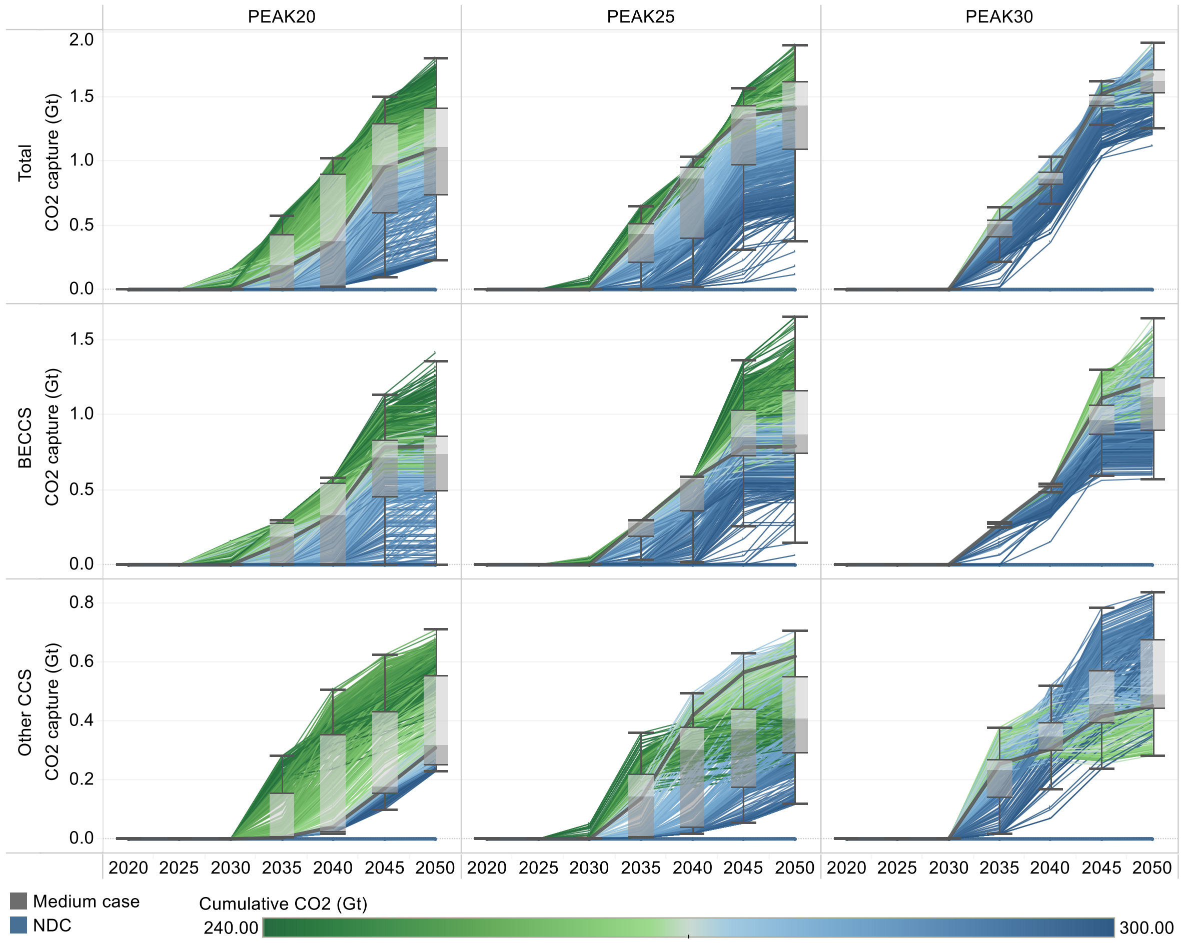

Supplementary Fig. 3. CO_2 capture by CCS technology (unit: Gt). The box plot shows the first quantile, intermediate range (IQR), and third quantile of all the results, where the data range within 1.5 times the IQR is denoted with whiskers. The thick blue line represents the pathway of the NDC scenario, and the thick grey line represents the pathway for the intermediate case in each scenario. The divergent colour from blue to green reflects the increasing stringency of the cumulative carbon budget. The cumulative CO_2 parameter corresponds to the absolute value of China's cumulative carbon budget for 2010-2050. BECCS = bioenergy with carbon capture and storage; CCS = carbon capture and storage.

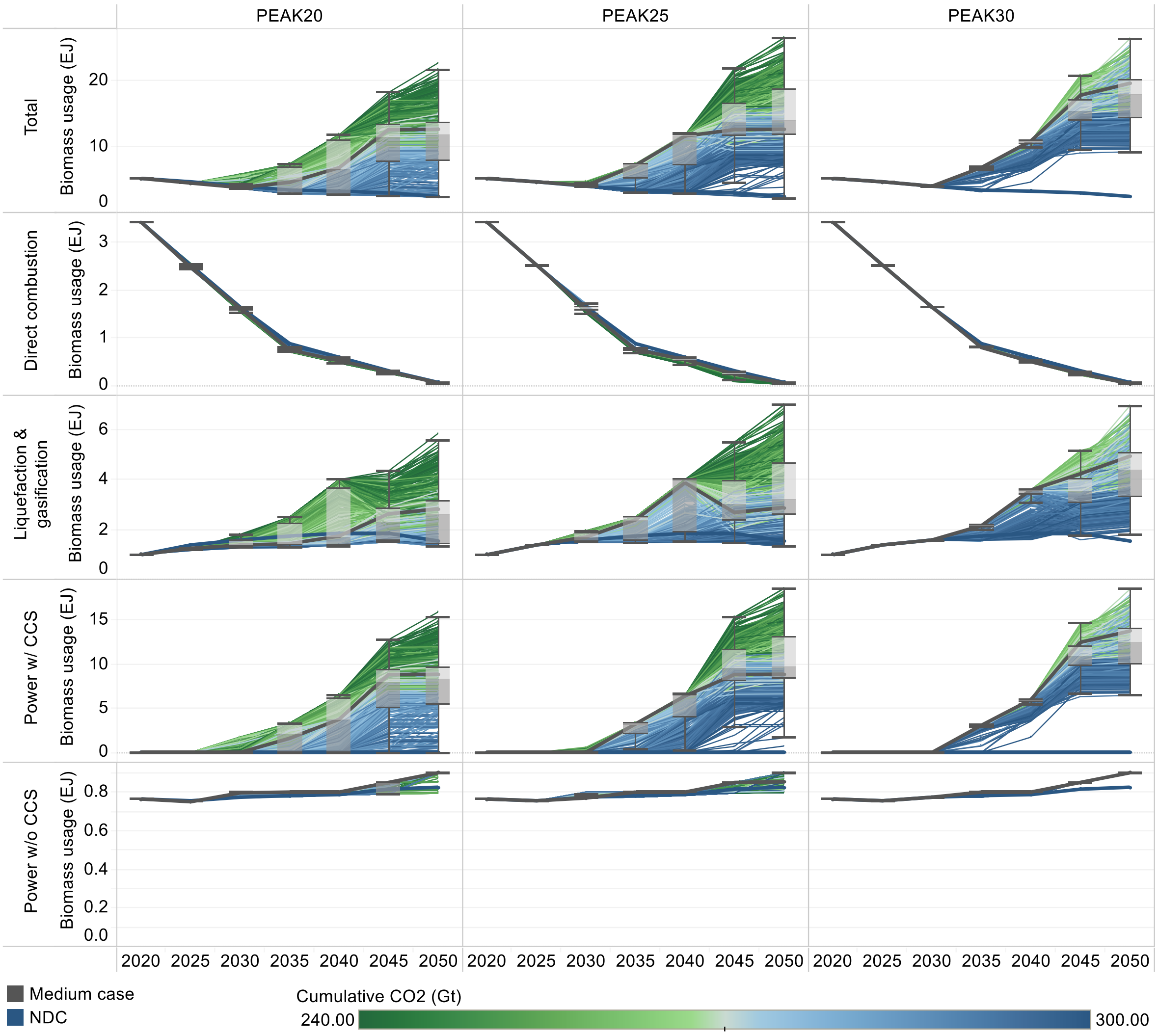

Supplementary Fig. 4. Biomass usage for different purposes. The box plot shows the first quantile, intermediate range (IQR), and third quantile of all the results, where the data range within 1.5 times the IQR is denoted with whiskers. The thick blue line represents the pathway of the NDC scenario, and the thick grey line represents the pathway for the intermediate case in each scenario. The divergent colour from blue to green reflects the increasing stringency of the cumulative carbon budget. The cumulative CO_2 parameter corresponds to the absolute value of China's cumulative carbon budget for 2010-2050. In this figure, w/ CCS means that this technology is equipped with carbon capture and storage, while w/o CCS means that it is not equipped.

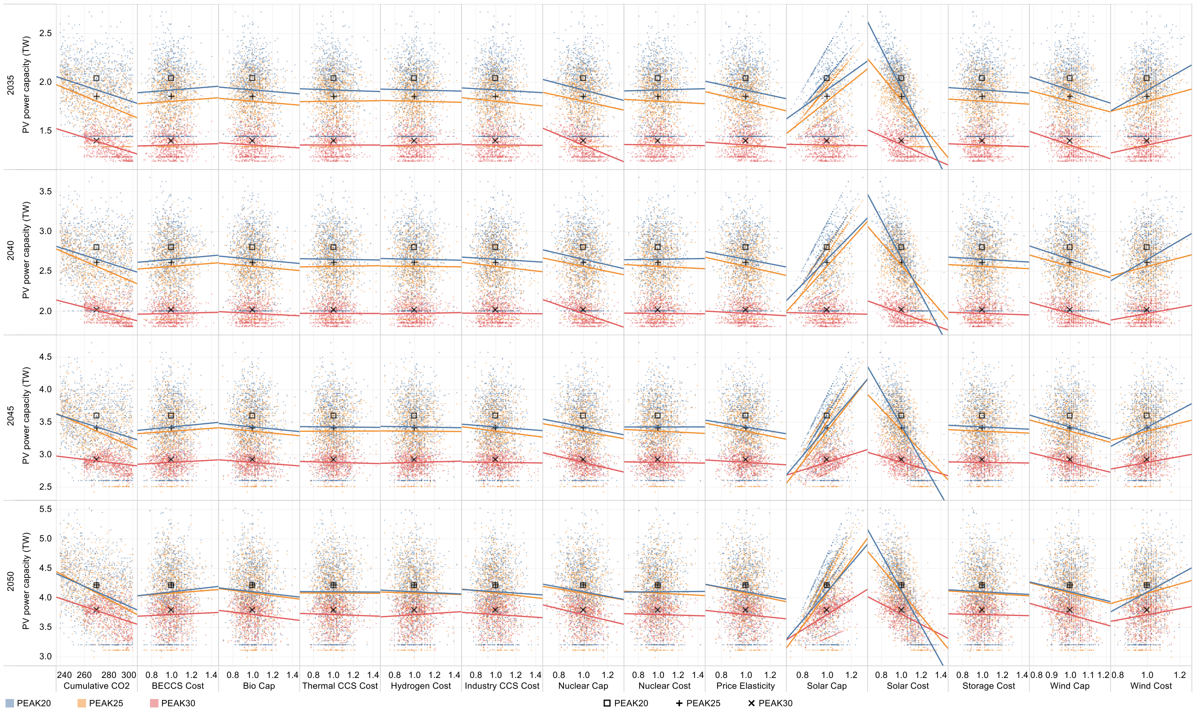

Supplementary Fig. 5. Scatter plot and linear regression results for the installed PV power capacity and uncertain Latin hypercube sampling-based variables. The intermediate cases of PEAK20, PEAK25, and PEAK30 are denoted with square, plus, and multiplication signs, respectively. PV = photovoltaic.

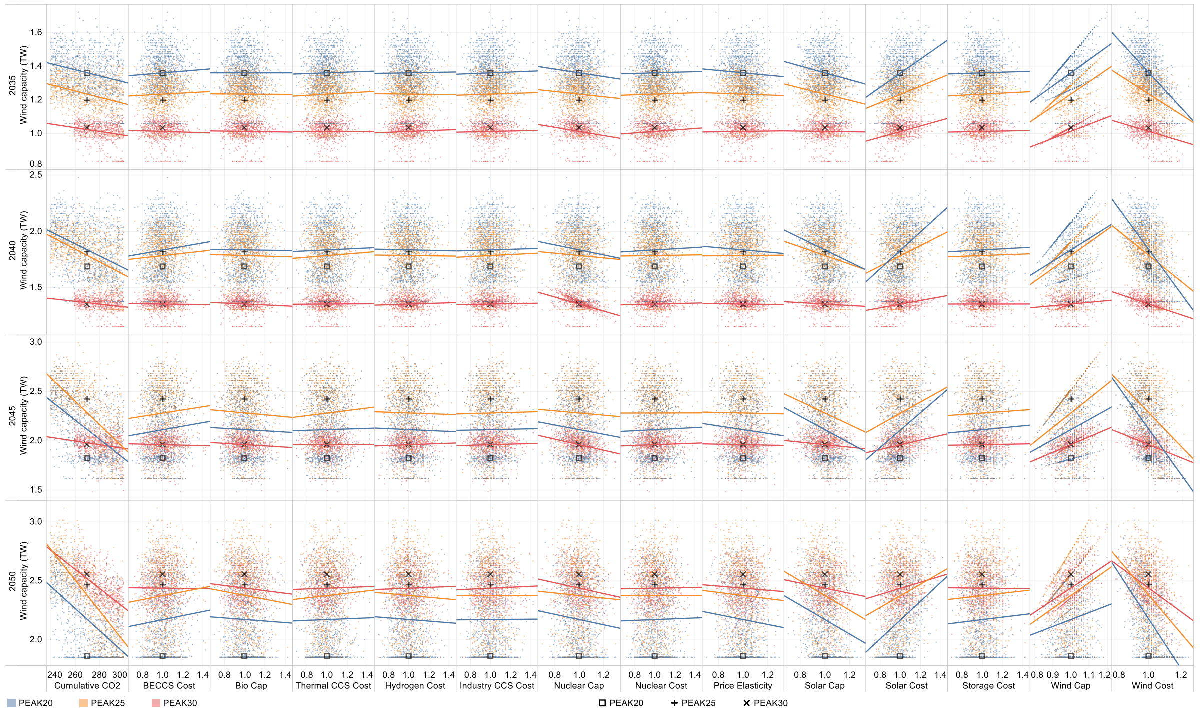

Supplementary Fig. 6. Scatter plot and linear regression results for the installed wind power capacity and uncertain Latin hypercube sampling-based variables. The intermediate cases for PEAK20, PEAK25, and PEAK30 are denoted with square, plus, and multiplication signs, respectively.

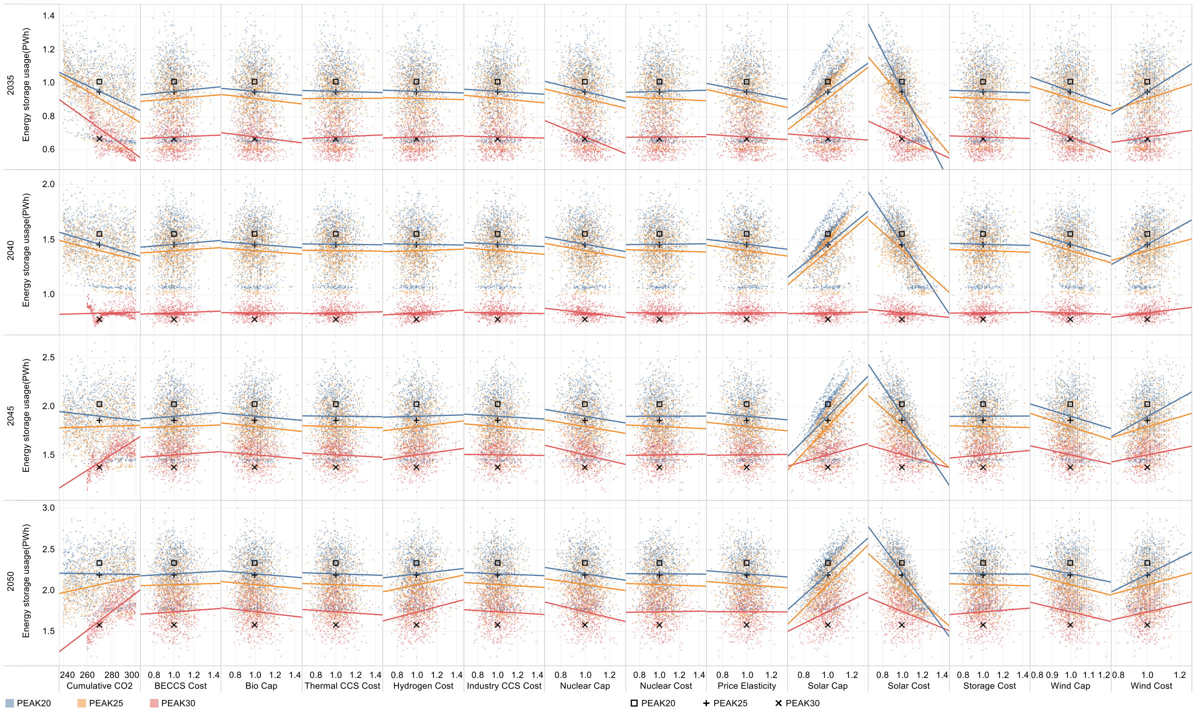

Supplementary Fig. 7. Scatter plot and linear regression results for the annual energy storage usage and uncertain Latin hypercube sampling-based variables. The intermediate cases for PEAK20, PEAK25, and PEAK30 are denoted with square, plus, and multiplication signs, respectively.

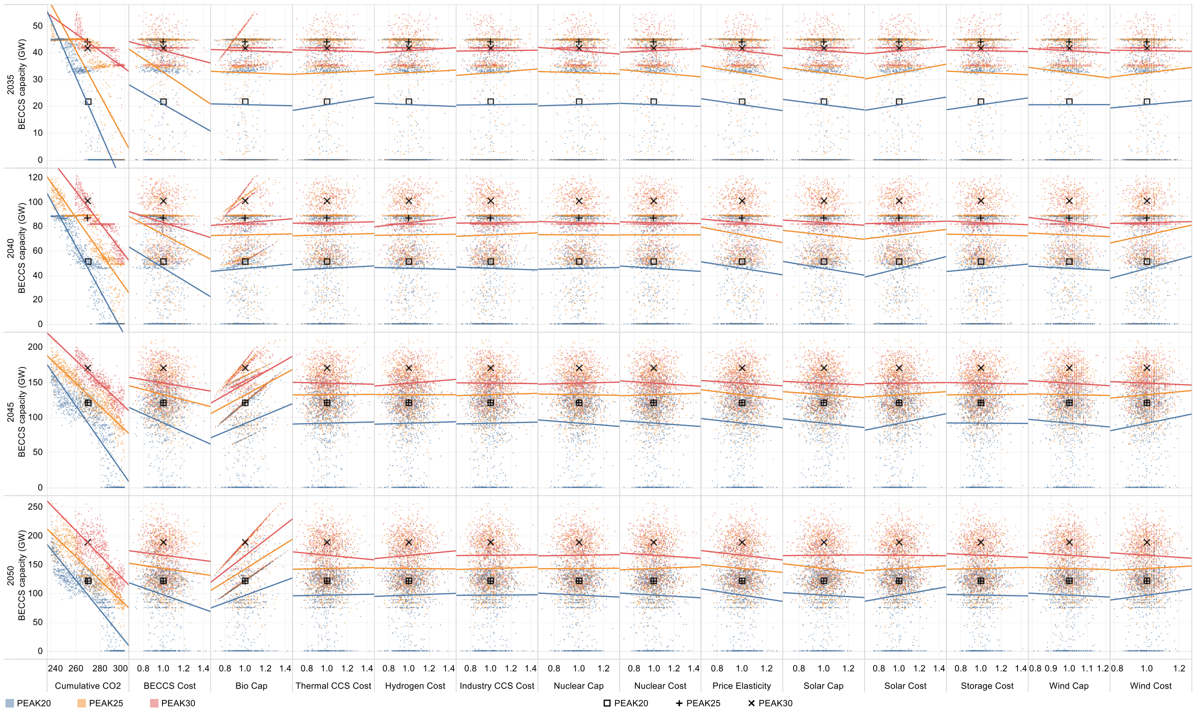

Supplementary Fig. 8. Scatter plot and linear regression results for the BECCS power plant capacity and uncertain Latin hypercube sampling-based variables. The intermediate cases for PEAK20, PEAK25, and PEAK30 are devoted by square, plus, and multiplication signs, respectively. BECCS = bioenergy with carbon capture and storage.

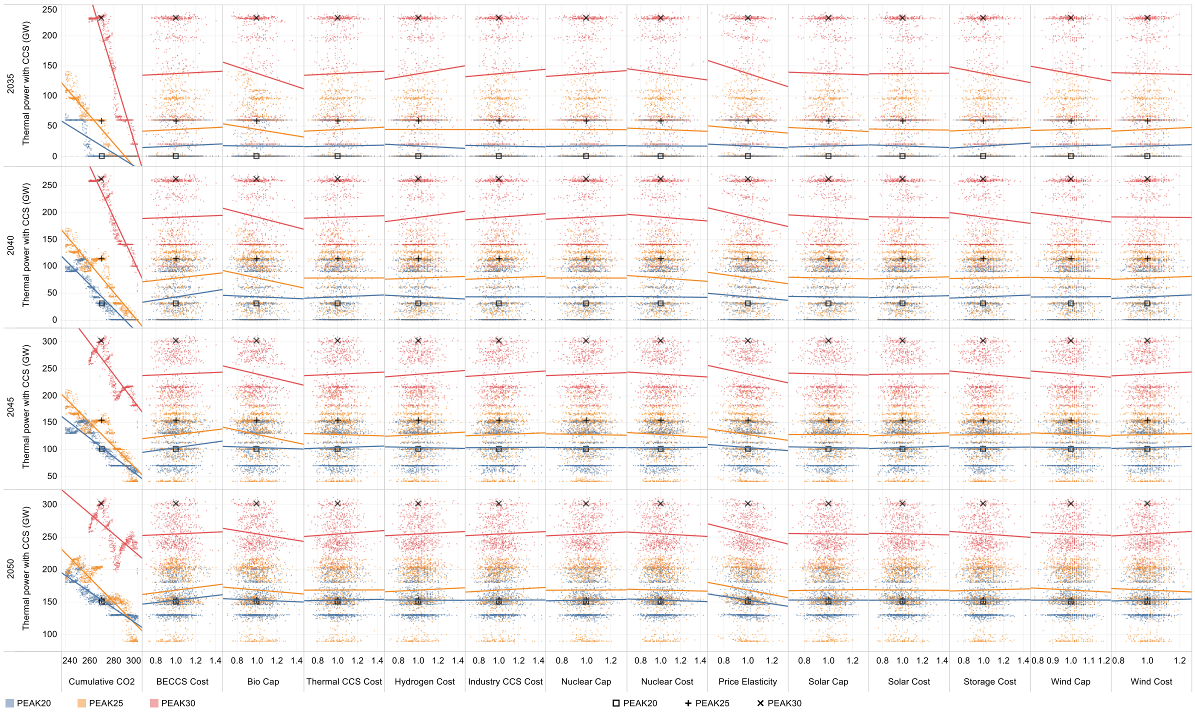

Supplementary Fig. 9. Scatter plot and linear regression results for the thermal power with CCS capacity and uncertain Latin hypercube sampling-based variables. The intermediate cases for PEAK20, PEAK25, and PEAK30 are denoted with square, plus, and multiplication signs, respectively. CCS = carbon capture and storage.

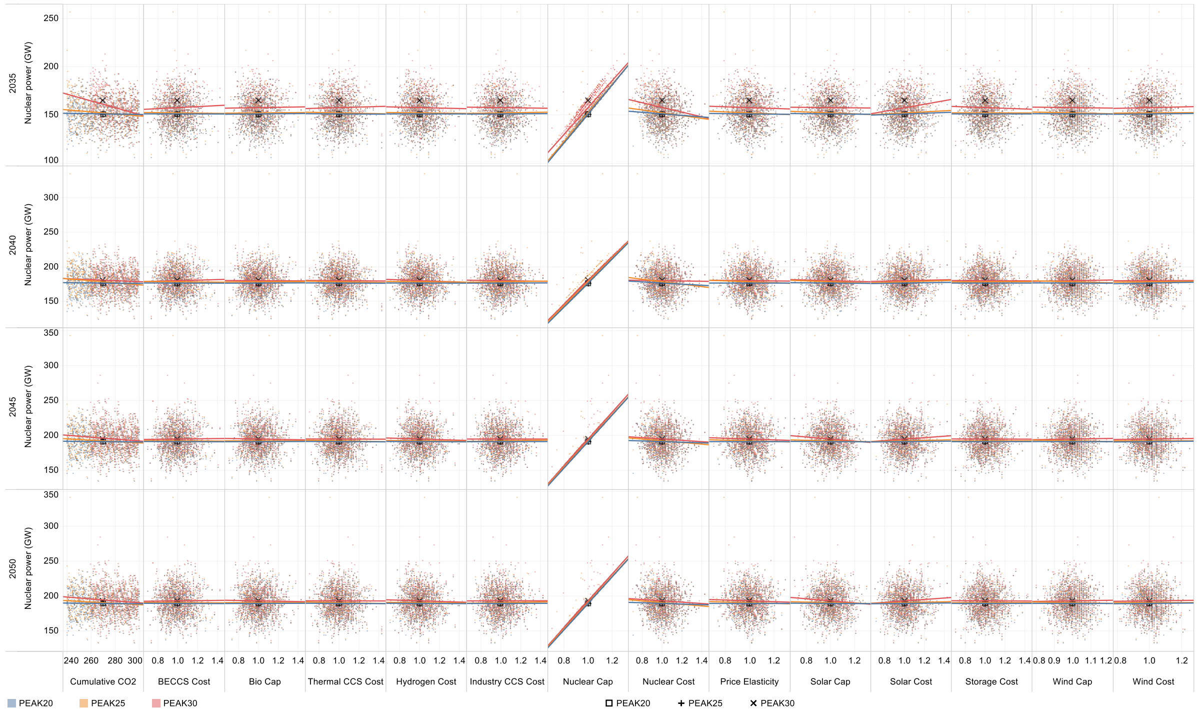

Supplementary Fig. 10. Scatter plot and linear regression results for the nuclear power capacity and uncertain Latin hypercube sampling-based variables. The intermediate cases for PEAK20, PEAK25, and PEAK30 are denoted with square, plus, and multiplication signs, respectively.

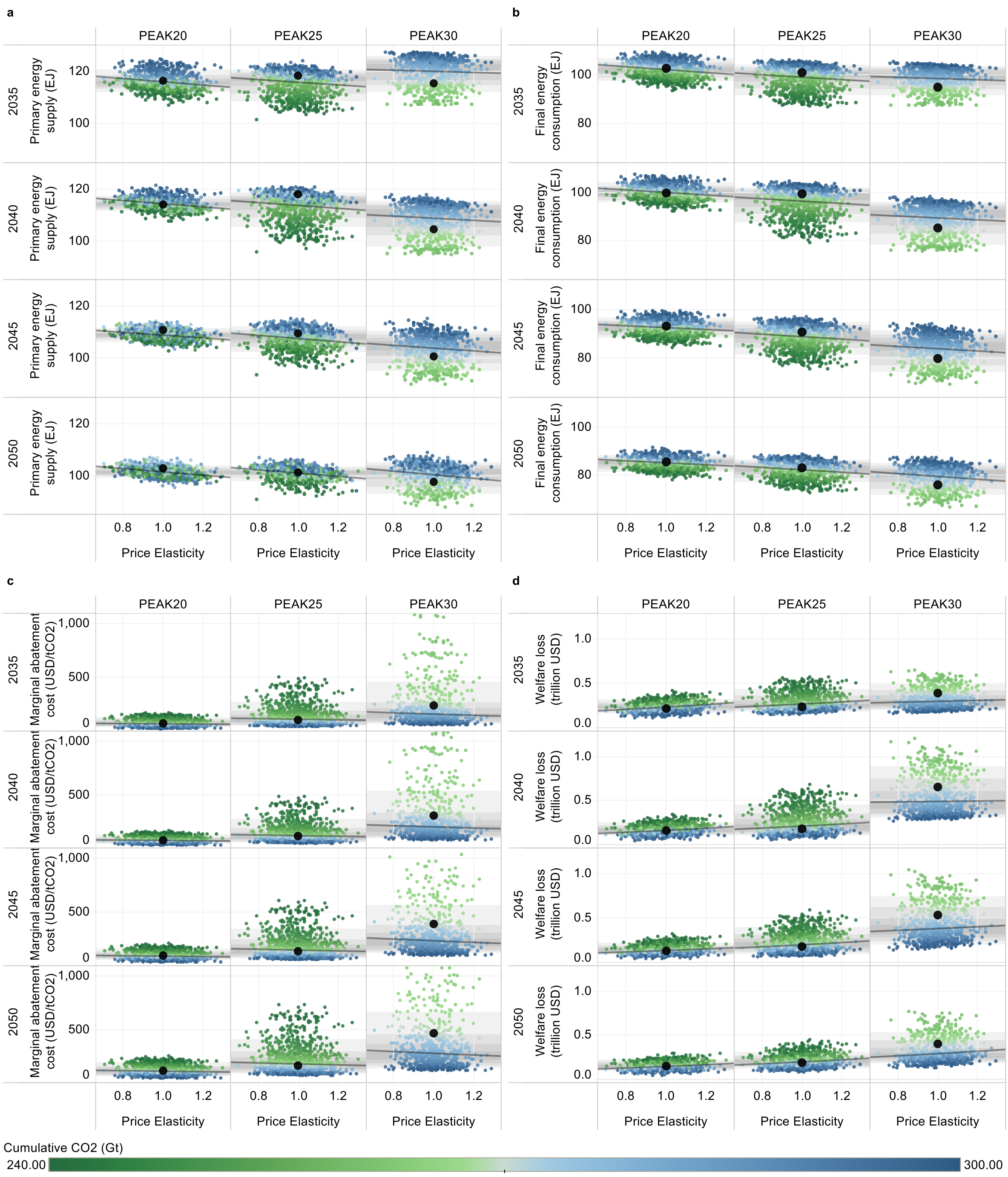

Supplementary Fig. 11. Scatter plot of the impacts of price elasticity on the primary energy supply, final energy consumption, marginal abatement cost and welfare loss. a Impact of price elasticity on primary energy supply. **b** Impact of price elasticity on final energy consumption. **c** Impact of price elasticity on marginal abatement cost. **d** Impact of price elasticity on welfare loss. The shadows of different levels indicate the positions of the ten quantiles of variables. The divergent colour from blue to green reflects the increasing stringency of the cumulative carbon budget. The cumulative CO_2 parameter corresponds to the absolute value of China's cumulative carbon budget for 2010-2050. Black dots represent the intermediate cases.

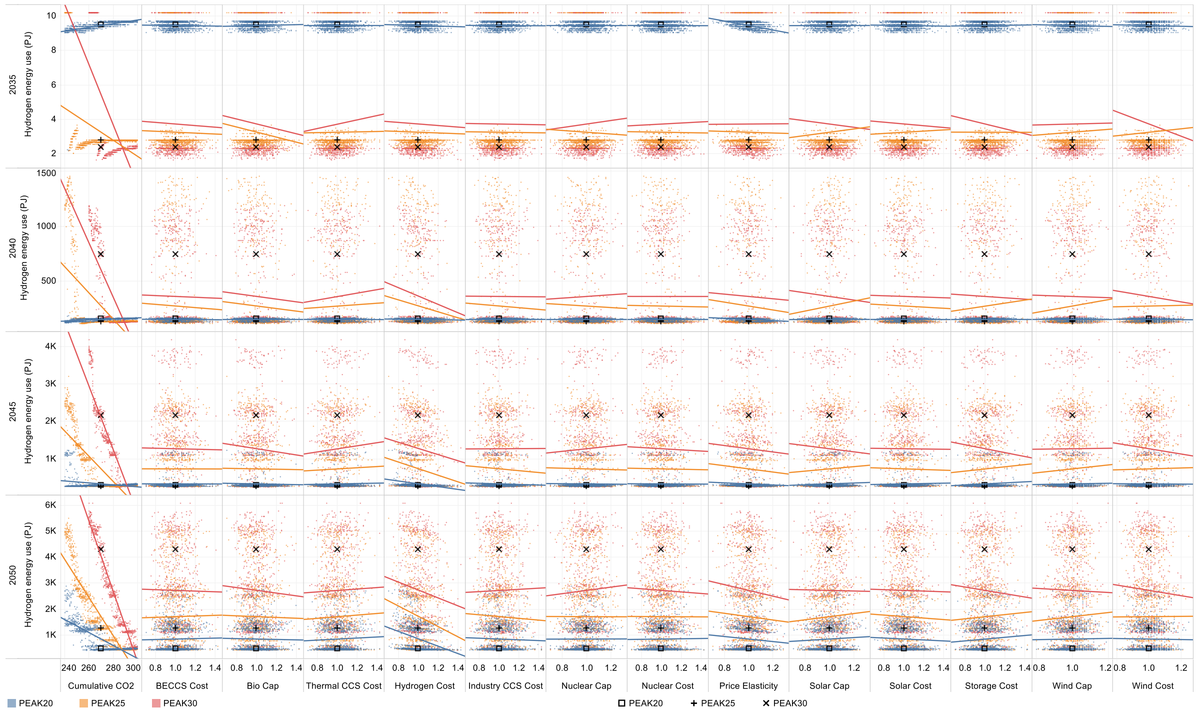

Supplementary Fig. 12. Scatter plot and linear regression results for the hydrogen energy use and uncertain Latin hypercube sampling-based variables. The intermediate cases for PEAK20, PEAK25, and PEAK30 are denoted with square, plus, and multiplication signs, respectively.

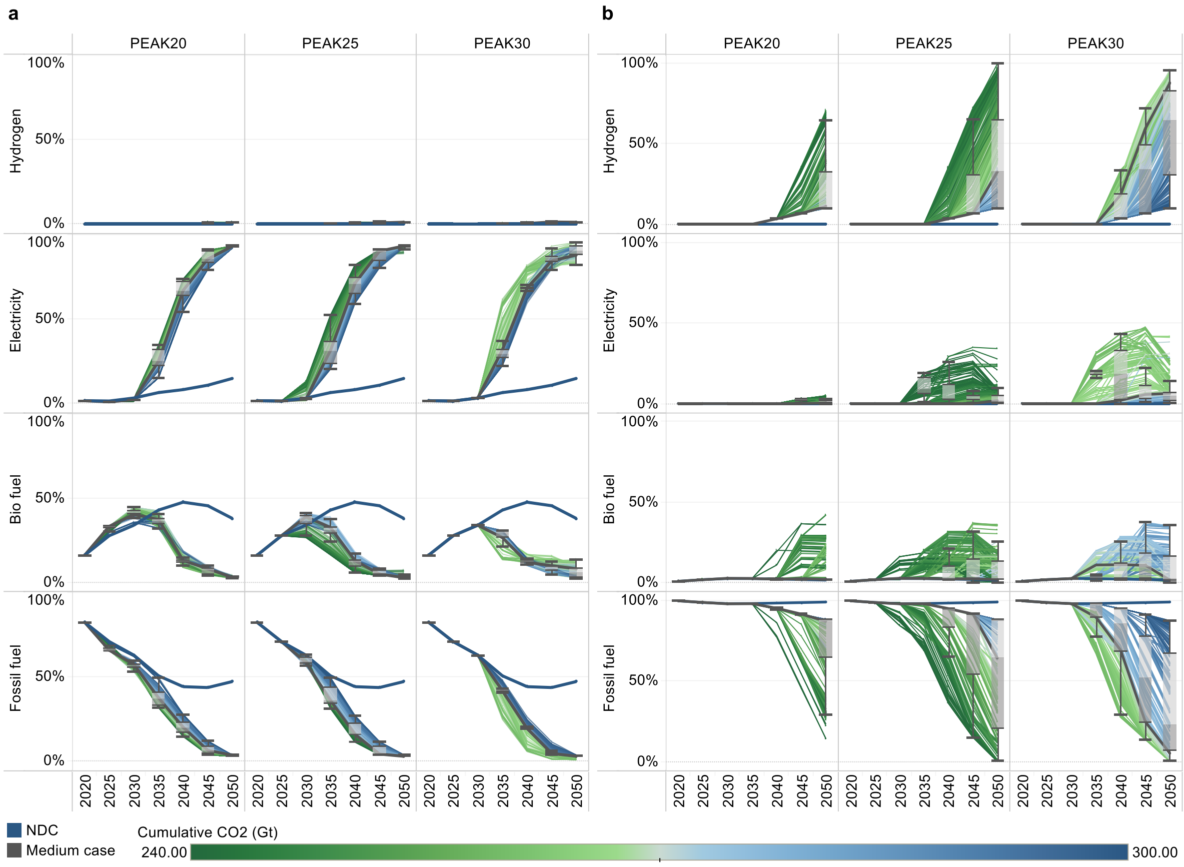

Supplementary Fig. 13. The fuel mix for road transport. a Road passenger transport. **b** Road freight transport. The box plot shows the first quantile, intermediate range (IQR), and third quantile of all the results, where the data range within 1.5 times the IQR is denoted with whiskers. The thick blue line represents the pathway of the NDC scenario, and the thick grey line represents the pathway for the intermediate case in each scenario. The divergent colour from blue to green reflects the increasing stringency of the cumulative carbon budget. The cumulative CO_2 parameter corresponds to the absolute value of China's cumulative carbon budget for 2010-2050.

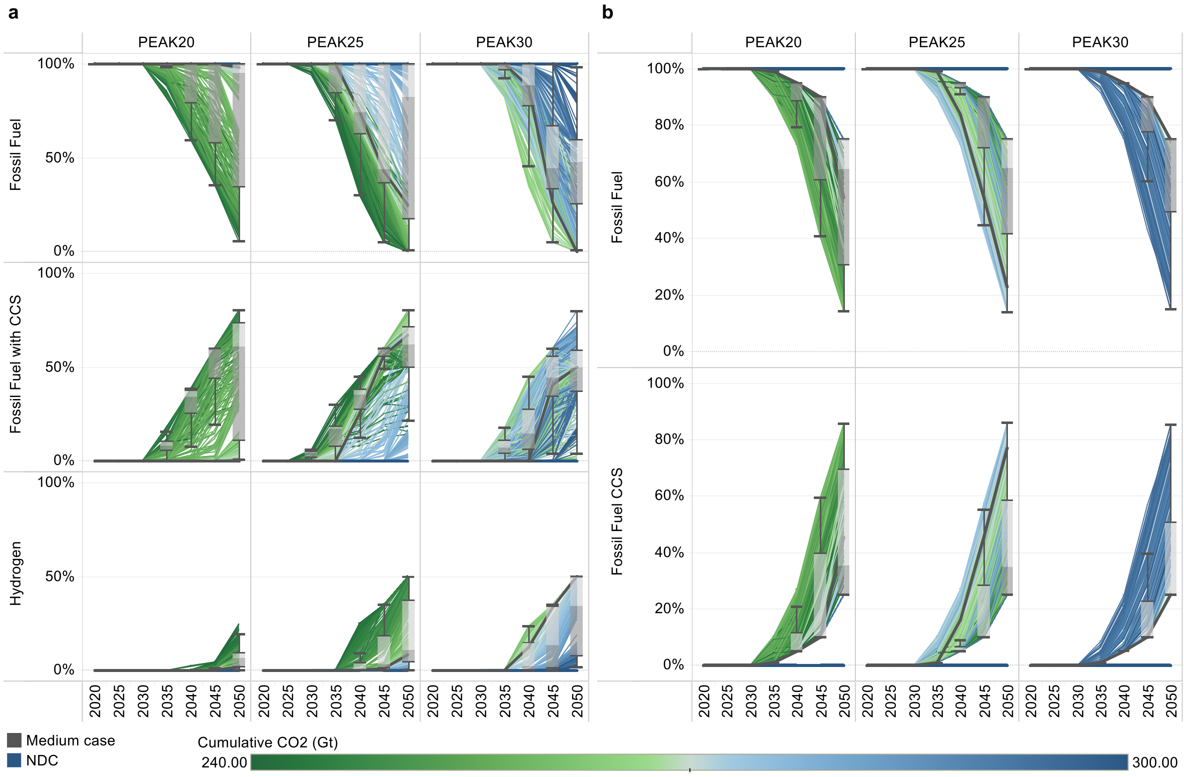

Supplementary Fig. 14. Production share of different technologies for iron and cement making. a Iron making. **b** Cement making. The box plot shows the first quantile, intermediate range (IQR), and third quantile of all the results, where the data range within 1.5 times the IQR is denoted with whiskers. The thick blue line represents the pathway of the NDC scenario, and the thick grey line represents the pathway for the intermediate case in each scenario. The divergent colour from blue to green reflects the increasing stringency of the cumulative carbon budget. The cumulative CO_2 parameter corresponds to the absolute value of China's cumulative carbon budget for 2010-2050. CCS = carbon capture and storage.

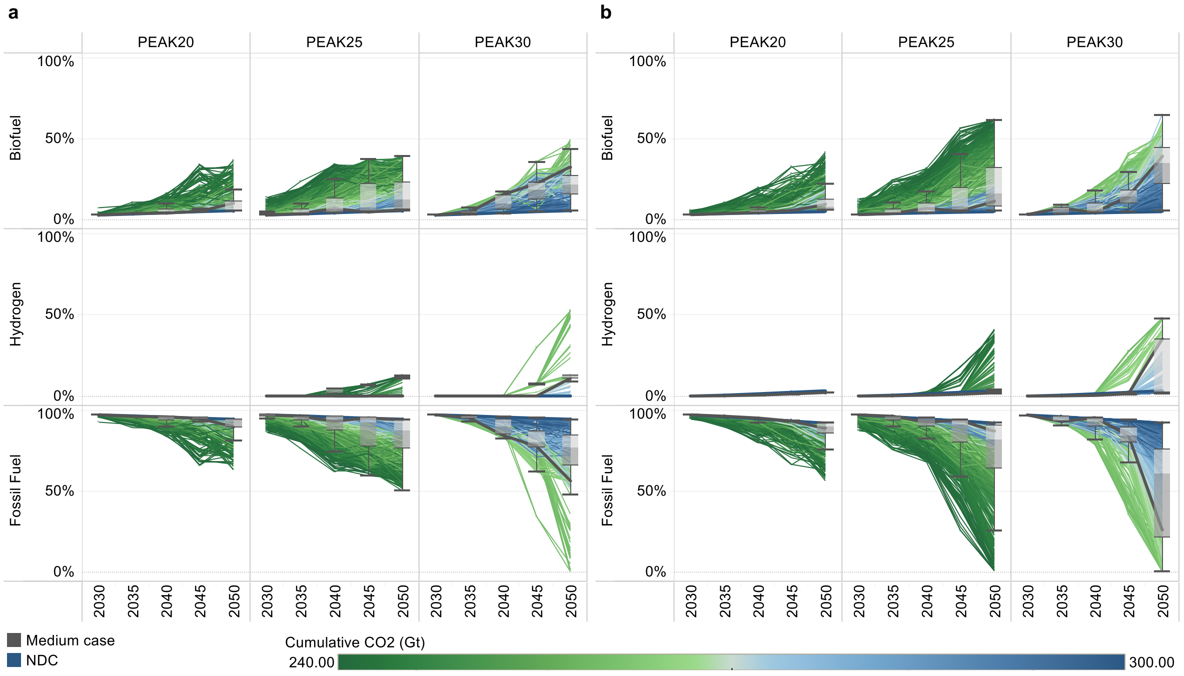

Supplementary Fig. 15. The fuel mix for air transport. a Passenger air transport. **b** Freight air transport. The box plot shows the first quantile, intermediate range (IQR), and third quantile of all the results, where the data range within 1.5 times the IQR is denoted with whiskers. The thick blue line represents the pathway of the NDC scenario, and the thick grey line represents the pathway for the intermediate case in each scenario. The divergent colour from blue to green reflects the increasing stringency of the cumulative carbon budget. The cumulative CO_2 parameter corresponds to the absolute value of China's cumulative carbon budget for 2010-2050.

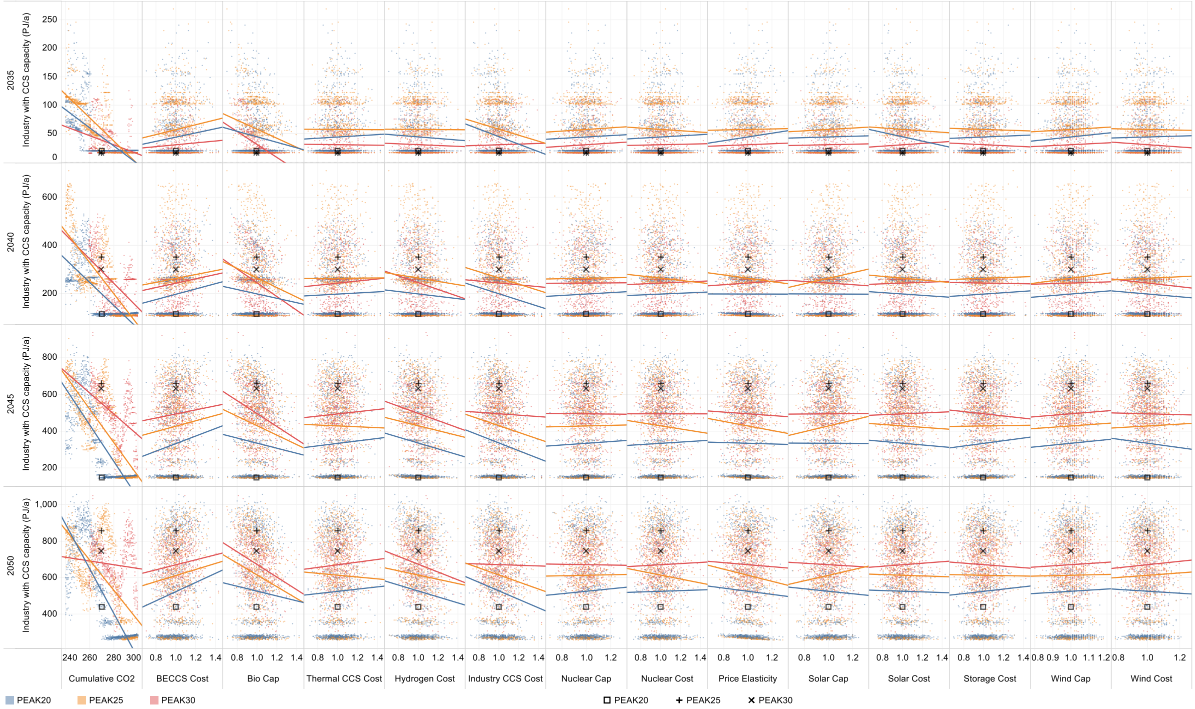

Supplementary Fig. 16. Scatter plot and linear regression results for the industry fossil fuel use with CCS and uncertain Latin hypercube sampling-based variables. The intermediate cases for PEAK20, PEAK25, and PEAK30 are denoted with square, plus, and multiplication signs, respectively. CCS = carbon capture and storage.

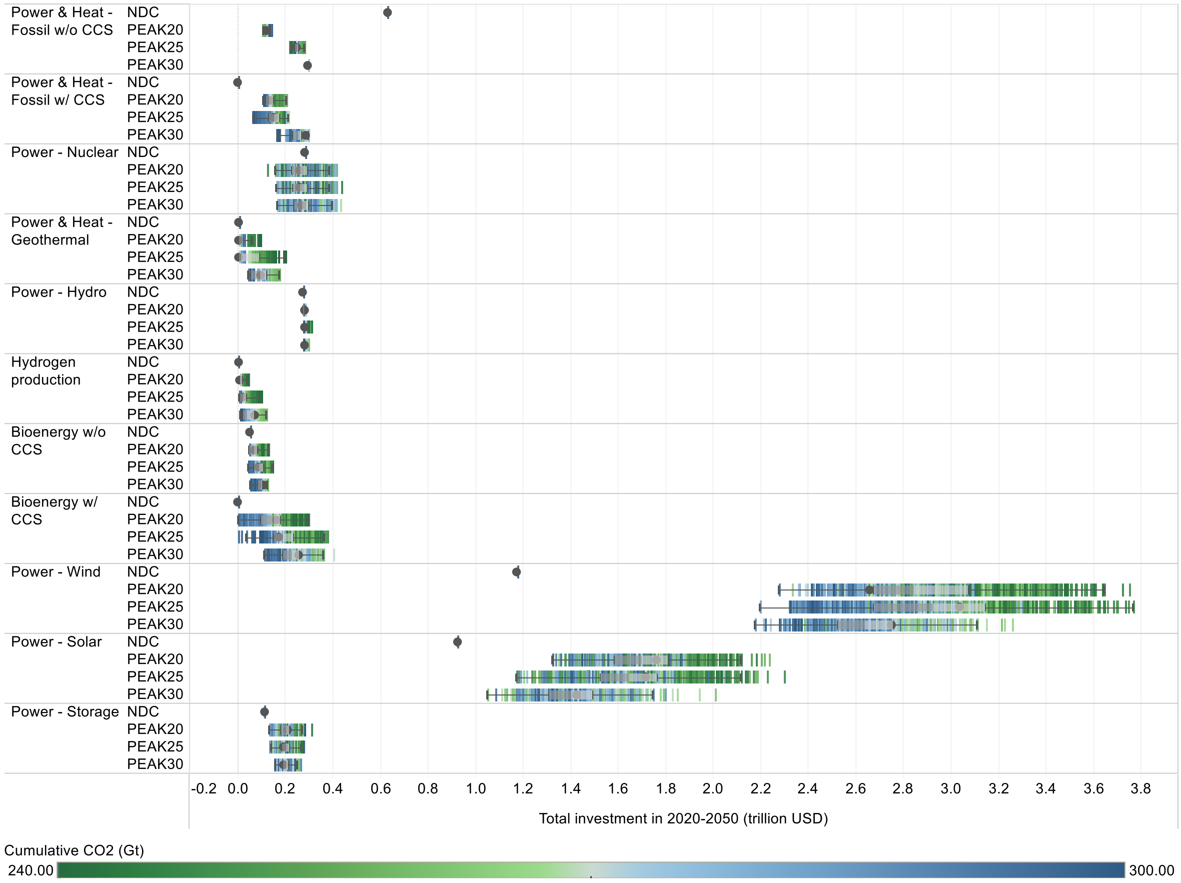

Supplementary Fig. 17. Total investment in different energy supply types in 2020-2050 (unit: trillion US dollars). The box plot shows the first quantile, intermediate range (IQR), and third quantile of all the results, where the data range within 1.5 times the IQR is denoted with whiskers. The black points are the investments for the intermediate case. The divergent colour from blue to green reflects the increasing stringency of the cumulative carbon budget. The cumulative CO_2 parameter corresponds to the absolute value of China's cumulative carbon budget for 2010-2050. In this figure, w/ CCS means that this technology is equipped with carbon capture and storage, while w/o CCS means that it is not equipped.

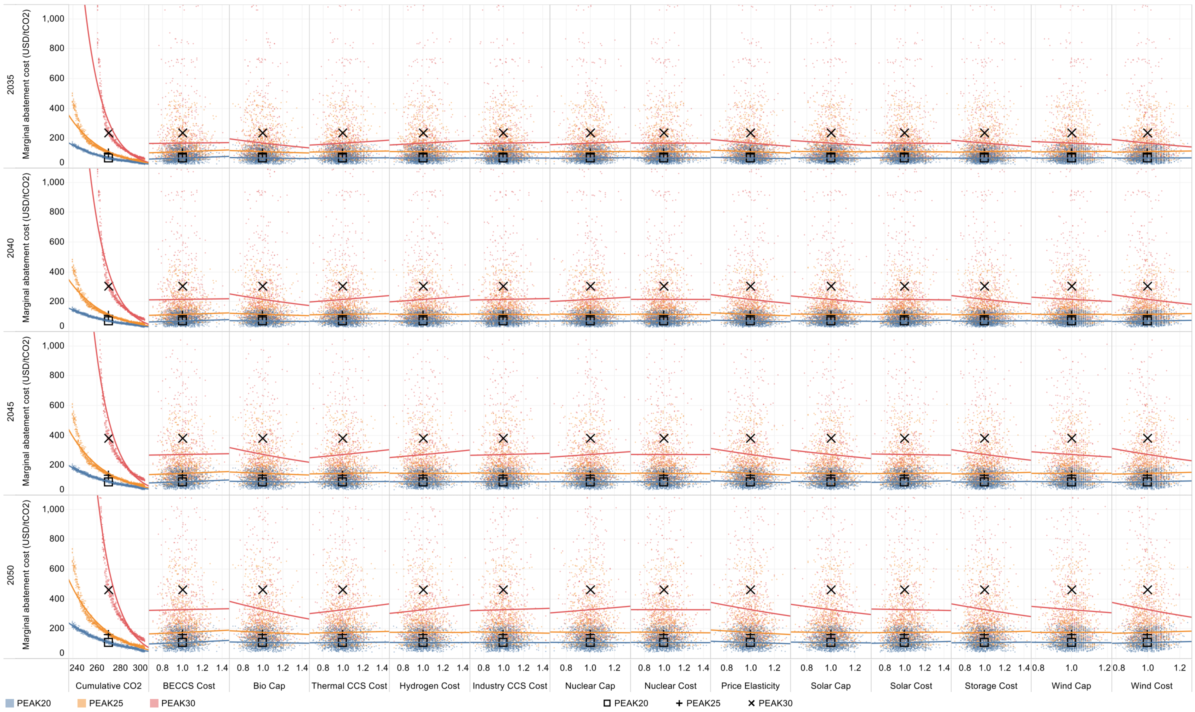

Supplementary Fig. 18. Scatter plot and exponential regression results for marginal abatement cost and uncertain Latin hypercube sampling-based variables. The intermediate cases for PEAK20, PEAK25, and PEAK30 are denoted with square, plus, and multiplication signs, respectively.

References

1. IEA. *Energy Technology Perspectives 2020*. (IEA, Paris, 2020).
2. CEC. *China Power Industry Annual Development Report 2020*. (China Building Materials Press, Beijing, 2020).
3. IEAGHG. *Towards Zero Emissions CCS In Power Plants Using Higher Capture Rates Of Biomass*. (IEAGHG, Cheltenham, 2019).
4. IEAGHG. *CCS In Energy And Climate Scenarios*. (IEAGHG, Cheltenham, 2019).
5. IEA. *World Energy Outlook 2020*. (IEA, Paris, 2020).
6. Zhang, P. *Evaluation of the Techno-Economics of Nuclear Hydrogen Production using HTGR (China)*. (IAEA-TECDOC--1859(COMPANION CD-ROM)). (International Atomic Energy Agency, Vienna, 2018).
7. IRENA. *Electricity Storage Valuation Framework: Assessing System Value and Ensuring Project Viability*. (International Renewable Energy Agency, Abu Dhabi, 2020).
8. IEA. *Projected Costs of Generating Electricity 2020*. (IEA, Paris, 2020).
9. Zhang, S. & Chen, W. Code and data for Nature Communications paper "Assessing the energy transition in China towards carbon neutrality with a probabilistic framework". Zenodo, doi:10.5281/zenodo.5717886 (2021).
